# Mobile phone signal exposure triggers a hormesis-like effect in *Atm*^+/+^ and *Atm*^−/−^ mouse embryonic fibroblasts

**DOI:** 10.1038/srep37423

**Published:** 2016-11-18

**Authors:** Chuan Sun, Xiaoxia Wei, Yue Fei, Liling Su, Xinyuan Zhao, Guangdi Chen, Zhengping Xu

**Affiliations:** 1 Bioelectromagnetics Laboratory, Zhejiang University School of Medicine, Hangzhou 310058, China; 2 Collaborative Innovation Center for Diagnosis and Treatment of Infectious Diseases, Zhejiang University, Hangzhou 310003, China; 3 Institute of Environmental Health, Zhejiang University School of Public Health, Hangzhou 310058, China

## Abstract

Radiofrequency electromagnetic fields (RF-EMFs) have been classified by the International Agency for Research on Cancer as possible carcinogens to humans; however, this conclusion is based on limited epidemiological findings and lacks solid support from experimental studies. In particular, there are no consistent data regarding the genotoxicity of RF-EMFs. Ataxia telangiectasia mutated (ATM) is recognised as a chief guardian of genomic stability. To address the debate on whether RF-EMFs are genotoxic, we compared the effects of 1,800 MHz RF-EMF exposure on genomic DNA in mouse embryonic fibroblasts (MEFs) with proficient (*Atm*^+/+^) or deficient (*Atm*^−/−^) ATM. In *Atm*^+/+^ MEFs, RF-EMF exposure for 1 h at an average special absorption rate of 4.0 W/kg induced significant DNA single-strand breaks (SSBs) and activated the SSB repair mechanism. This effect reduced the DNA damage to less than that of the background level after 36 hours of exposure. In the *Atm*^−/−^ MEFs, the same RF-EMF exposure for 12 h induced both SSBs and double-strand breaks and activated the two repair processes, which also reduced the DNA damage to less than the control level after prolonged exposure. The observed phenomenon is similar to the hormesis of a toxic substance at a low dose. To the best of our knowledge, this study is the first to report a hormesis-like effect of an RF-EMF.

With the rapid development of mobile communication, public concerns have been raised about possible health hazards induced by exposure to the radiofrequency electromagnetic fields (RF-EMFs) emitted by mobile phones^1^. Although large-scale *in vivo* and *in vitro* studies have investigated the biological and health effects of RF-EMFs, the results are contradictory^1^,^2^. Based on limited epidemiological evidence indicating an association of mobile phone use with an increased risk of glioma, the International Agency for Research on Cancer (IARC) has classified RF-EMFs as possibly carcinogenic to humans^3^. However, owing to a lack of consistent experimental support, it remains unclear whether there is a causal relationship between RF-EMF exposure and tumour development.

Genotoxicity is the gold standard used to judge whether a substance is carcinogenic. Thus, the effects of RF-EMFs on DNA have been extensively assessed in various cellular and animal models. Unfortunately, currently available data are inconsistent or difficult to compare^4^,^5^,^6^. Possible causes of this inconsistency are that different groups 1) adopted different biological models, 2) applied different exposure systems and/or exposure parameters, and/or 3) employed different protocols to detect the same endpoint. To overcome these concerns, our group proposed the adoption of a systematic approach to faithfully elucidate the effects of RF-EMFs, namely, using the same exposure system and parameters by a same group of researchers to explore the same biological effects in various biological systems. To reveal the influence of biological models on RF-EMF genotoxicity, we have employed an internationally well-accepted exposure system and the γH2AX focus formation assay to compare the effects of 1,800 MHz RF-EMF exposure on DNA damage in six different cell types. Our data show that different cells respond differently to the exposure, and the exposure only induces γH2AX focus formation in human skin fibroblasts and Chinese hamster lung fibroblasts^7^. However, the slight increase in DNA damage does not result in significant DNA fragmentation or abnormal cellular behaviour, suggesting that the RF-EMF-induced DNA damage might be repaired or compensated for by the cells^7^. These findings prompted us to adopt a biological system with a deficiency in DNA repair to more sensitively and more accurately reflect the impact of RF-EMF exposure on genome stability.

Upon exposure to ionising irradiation or other DNA-damaging agents, ataxia telangiectasia mutated (ATM) is immediately activated through autophosphorylation, which is critical for the initiation of the DNA repair process^8^. ATM deficiency leads to inherent destabilisation of chromosomal integrity^8^, and ATM-deficient cells and animals are more sensitive to ionising irradiation^9^,^10^, oxidative stress^11^,^12^,^13^, and other insults^14^,^15^. Therefore, we used *Atm*-proficient (*Atm*^+/+^) and *Atm*-deficient (*Atm*^−/−^) mouse embryonic fibroblasts (MEFs) to explore the effects of 1,800 MHz RF-EMF exposure on cellular genomic DNA. To comprehensively evaluate such effects, we simultaneously employed alkaline and neutral comet assays to detect whether DNA single-strand breaks (SSBs) or double-strand breaks (DSBs) occurred^16^, and we examined whether the corresponding DNA damage repair pathways were activated. Furthermore, we observed whether the cell behaviour changed. Our results imply the existence of a hormesis-like effect in the cells in response to RF-EMF exposure.

## Results

### RF-EMF first induces and then decreases DNA damage in both *Atm* ^+/+^ and *Atm* ^−/−^ MEFs

**Figure 1:**
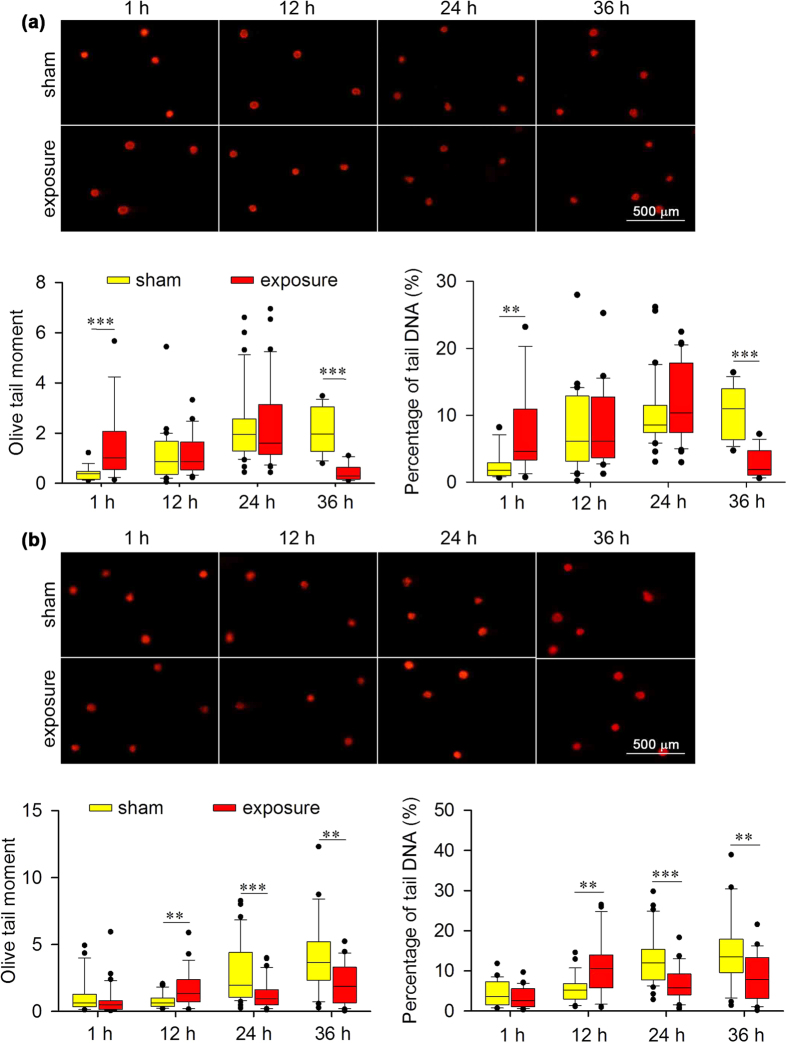
Effects of 1,800 MHz RF-EMF exposure on DNA fragmentation (alkaline comet assay) in *Atm*^+/+^ and *Atm*^−/−^ MEFs. Representative images show DNA fragmentation in (**a**) *Atm*^+/+^ and (**b**) *Atm*^−/−^ MEFs after sham exposure or exposure to 1,800 MHz RF-EMF at 4.0 W/kg for up to 36 h. Boxplots show the data of Olive tail moment and percentage of tail DNA (%); values were quantified in 18–24 fields of view, with each field containing 10–20 cells. The experiments were repeated at least 3 times. The Mann-Whitney rank-sum test was applied to determine the statistical significance of differences between the RF-EMF and sham exposure groups under the same experimental conditions. A probability level of *P* < 0.05 was considered statistically significant. **P < 0.01, ***P < 0.001.

To reveal the influence of 1,800 MHz RF-EMF exposure on cellular DNA, we applied the alkaline comet assay to examine DNA fragmentation status after RF-EMF exposure at an average special absorption rate (SAR) of 4.0 W/kg. In *Atm*^+/+^ MEFs, significant DNA fragmentation was observed after 1 hour (h) of exposure (Fig. 1a), suggesting that RF-EMF can induce DNA damage. Subsequently, we extended the exposure time up to 36 h to determine the time-dependent effects. No obvious changes occurred in the 12- or 24-h-exposed groups, and the levels of DNA fragmentation were even lower than the background level after 36 h of exposure (Fig. 1a). This phenomenon was also detected in the *Atm*^−/−^ MEFs; namely, DNA fragmentation increased after 12 h of exposure but decreased to lower than the control level after 24 and 36 h of exposure (Fig. 1b).

### RF-EMF induces transient DNA DSBs in *Atm* ^−/−^ MEFs but not *Atm* ^+/+^ MEFs

**Figure 2:**
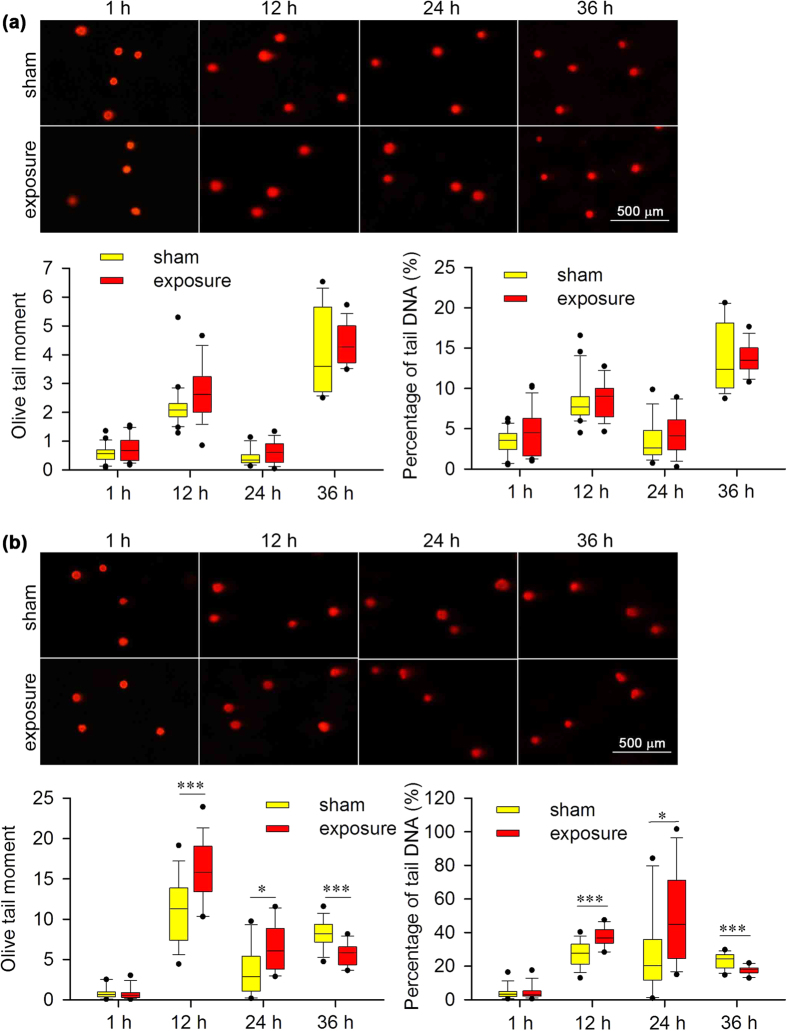
Effects of 1,800 MHz RF-EMF exposure on DNA fragmentation (neutral comet assay) in *Atm*^+/+^ and *Atm*^−/−^ MEFs. Representative images show DNA fragmentation in (**a**) *Atm*^+/+^ and (**b**) *Atm*^−/−^ MEFs after sham exposure or exposure to 1,800 MHz RF-EMF at 4.0 W/kg for up to 36 h. Boxplots show the data of Olive tail moment and percentage of tail DNA (%); values were quantified in 18–24 fields of view, with each field containing 10–20 cells. The experiments were repeated at least 3 times. The Mann-Whitney rank-sum test was applied to determine the statistical significance of differences between the RF-EMF and sham exposure groups under the same experimental conditions. A probability level of *P* < 0.05 was considered statistically significant. *P < 0.05, ***P < 0.001.

To determine what type of DNA damage was induced by RF-EMF exposure, we further applied the neutral comet assay to visualise whether DSBs occurred in either MEF type. RF-EMF exposure induced significant DNA fragmentation only in *Atm*^−/−^ MEFs after 12 and 24 h of exposure (Fig. 2), suggesting a lack of obvious DSBs in *Atm*^+/+^ MEFs. Similar to the phenomenon observed in the alkaline comet assay, DSBs in *Atm*^−/−^ MEFs were also decreased to levels lower than the background level after 36 h of exposure (Fig. 2b).

### RF-EMF activates the SSB repair pathway in *Atm* ^+/+^ MEFs and both the SSB and DSB repair systems in *Atm* ^−/−^ MEFs

**Figure 3:**
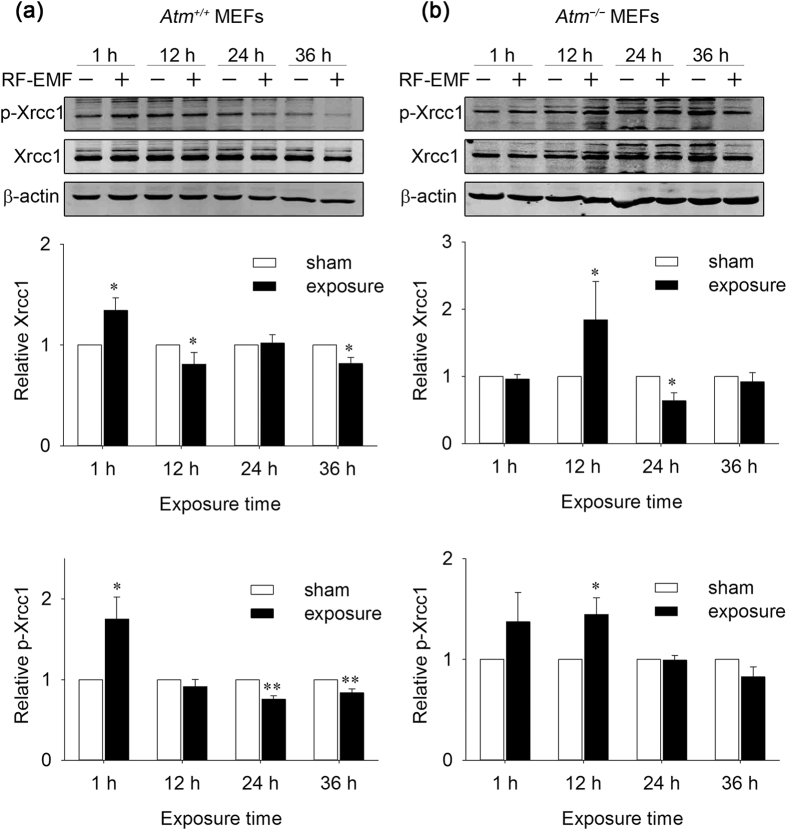
Effects of 1,800 MHz RF-EMF exposure on X-ray repair cross-complementing protein 1 (Xrcc1) expression and phosphorylation in *Atm*^+/+^ and *Atm*^−/−^ MEFs. Representative Western blot images show the Xrcc1 and phospho-Xrcc1 (p-Xrcc1) levels in *Atm*^+/+^ and *Atm*^−/−^ MEFs after sham exposure or exposure to 1,800 MHz RF-EMF at 4.0 W/kg for up to 36 h. To perform quantification analysis, the loading amounts of both Xrcc1 and p-Xrcc1 were normalised to β-actin. Values are shown as the means ± SEM; n = 3–4. The Mann-Whitney rank-sum test was applied to determine the statistical significance of differences between the RF-EMF and sham exposure groups under the same experimental conditions. A probability level of *P* < 0.05 was considered statistically significant. *P < 0.05, **P < 0.01. More details are available in the supplemental files.

**Figure 4:**
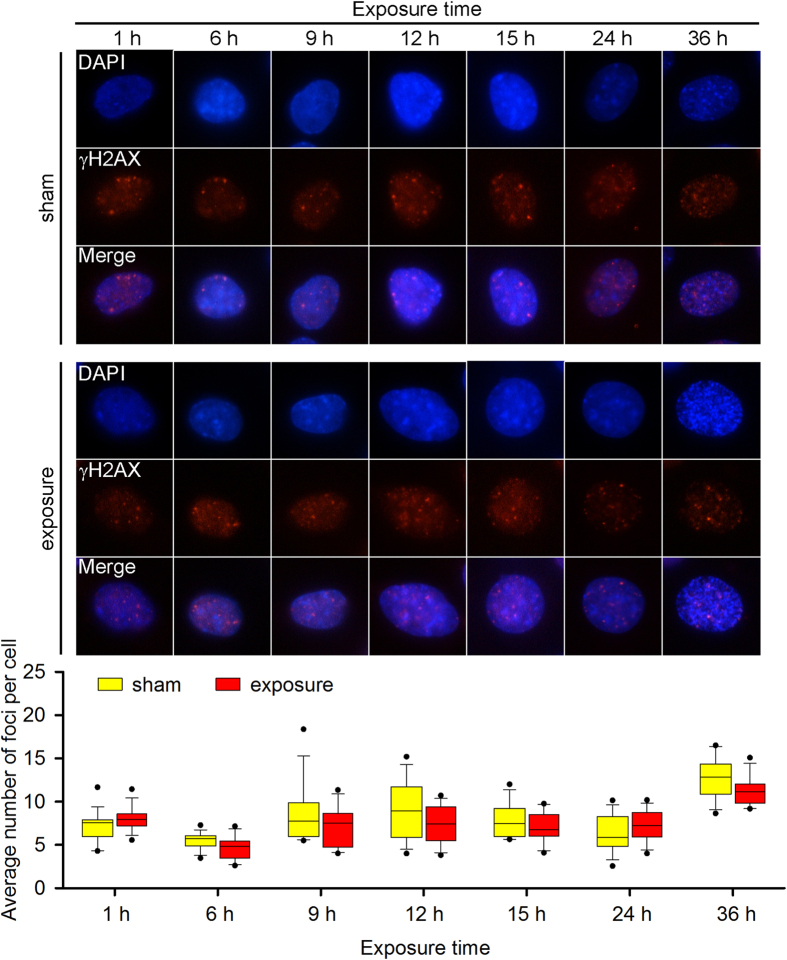
The effect of 1,800 MHz RF-EMF exposure on γH2AX focus formation in *Atm*^+/+^ MEFs. *Atm*^+/+^ MEFs were exposed to 4.0 W/kg 1,800 MHz RF-EMF for up to 36 h. A boxplot shows the average number of γH2AX foci per cell in each group; values were quantified in 12–18 fields of view, with each field containing 10–20 cells. The experiments were repeated at least 3 times. The Mann-Whitney rank-sum test was applied to determine the statistical significance of differences between the RF-EMF and sham exposure groups under the same experimental conditions. A probability level of *P* < 0.05 was considered statistically significant.

**Figure 5:**
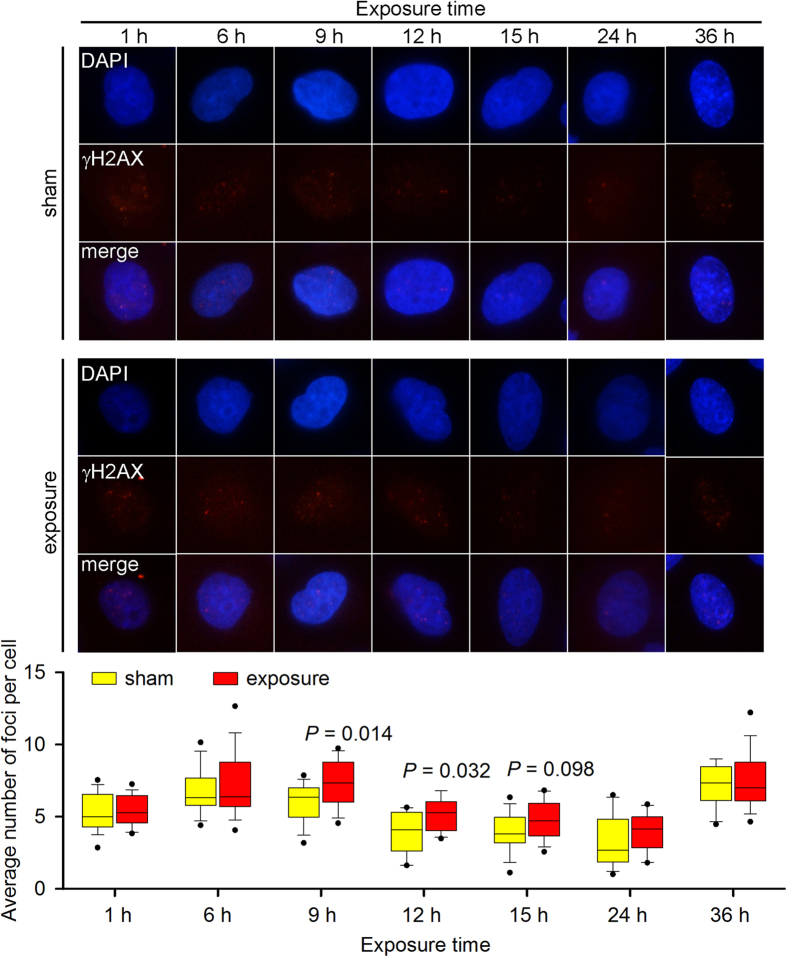
The effect of 1,800 MHz RF-EMF exposure on γH2AX focus formation in *Atm*^−/−^MEFs. *Atm*^**−/−**^ MEFs were exposed to 4.0 W/kg 1,800 MHz RF-EMF for up to 36 h. A boxplot shows the average number of γH2AX foci per cell in each group; values were quantified in 12–18 fields of view, with each field containing 10–20 cells. The experiments were repeated at least 3 times. The Mann-Whitney rank-sum test was applied to determine the statistical significance of differences between the RF-EMF and sham exposure groups under the same experimental conditions. A probability level of *P* < 0.05 was considered statistically significant.

To maintain genomic stability, cells have developed a series of DNA damage repair mechanisms to fix DNA breaks. X-ray repair cross-complementing protein 1 (Xrcc1) is critical in the process of SSB repair^17^, and γH2AX focus formation is an early biomarker of DSB repair^18^. To visualise potential activation of the corresponding DNA damage repair mechanism, we detected both markers in exposed cells by Western blotting and immunofluorescence staining. Xrcc1 phosphorylation and expression were increased in both *Atm*^+/+^ and *Atm*^−/−^ MEFs in response to the increased DNA damage (Fig. 3 and Supplementary Figs S1 and S2), suggesting activation of the SSB repair pathway. In addition, γH2AX foci levels were only elevated in the 9- and 12-h-exposed *Atm*^−/−^ MEFs (Figs 4 and 5), indicating that the DSB repair pathway was also activated in these cells.

### RF-EMF does not change cell cycle progression, viability, cell proliferation or apoptosis in either *Atm* ^+/+^ or *Atm* ^−/−^ MEFs

**Figure 6:**
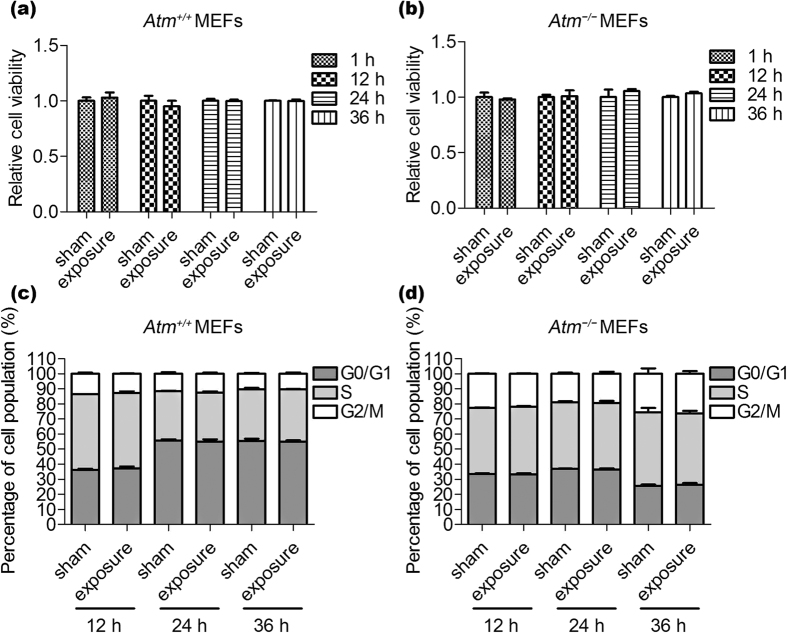
Effects of 1,800 MHz RF-EMF exposure on cell viability and cell cycle progression in *Atm*^+/+^ and *Atm*^−/−^ MEFs. (**a**,**b**) Relative cell viabilities in *Atm*^+/+^ and *Atm*^−/−^ MEFs after sham exposure or exposure to 1,800 MHz RF-EMF at 4.0 W/kg for 1, 12, 24, and 36 h. (**c**,**d**) Cell cycle distribution in *Atm*^+/+^ and *Atm*^−/−^ MEFs after sham exposure or exposure to 1,800 MHz RF-EMF at 4.0 W/kg for 12, 24, and 36 h. Values are shown as the mean ± SEM; n = 3–5. The two-tailed paired Student’s *t*-test was used to determine the statistical significance between the RF-EMF and sham exposure groups under the same experimental conditions. A probability level of *P* < 0.05 was considered statistically significant.

Cellular DNA damage usually leads to cell cycle arrest and cell growth inhibition. Thus, we examined whether RF-EMF affected these two factors. Exposure to RF-EMF for up to 36 h had no significant influence on the cell cycle or cell viability (Fig. 6). To exclude interference from the cell growth or death rates on the results, we simultaneously counted cell numbers, measured 5-ethynyl-2′-deoxyuridine (EdU) incorporation, and detected cell apoptosis and found that there were no significant changes (Supplementary Figs S3–S5). These data suggest that both *Atm*^+/+^ and *Atm*^−/−^ MEFs are able to compensate for the damage induced by RF-EMF exposure.

### The effects of 1,800 MHz RF-EMF exposure on MEFs are similar to the effects of low-dose 4-nitroquinoline 1-oxide (4NQO) and hydrogen peroxide (H_2_O_2_)

**Figure 7:**
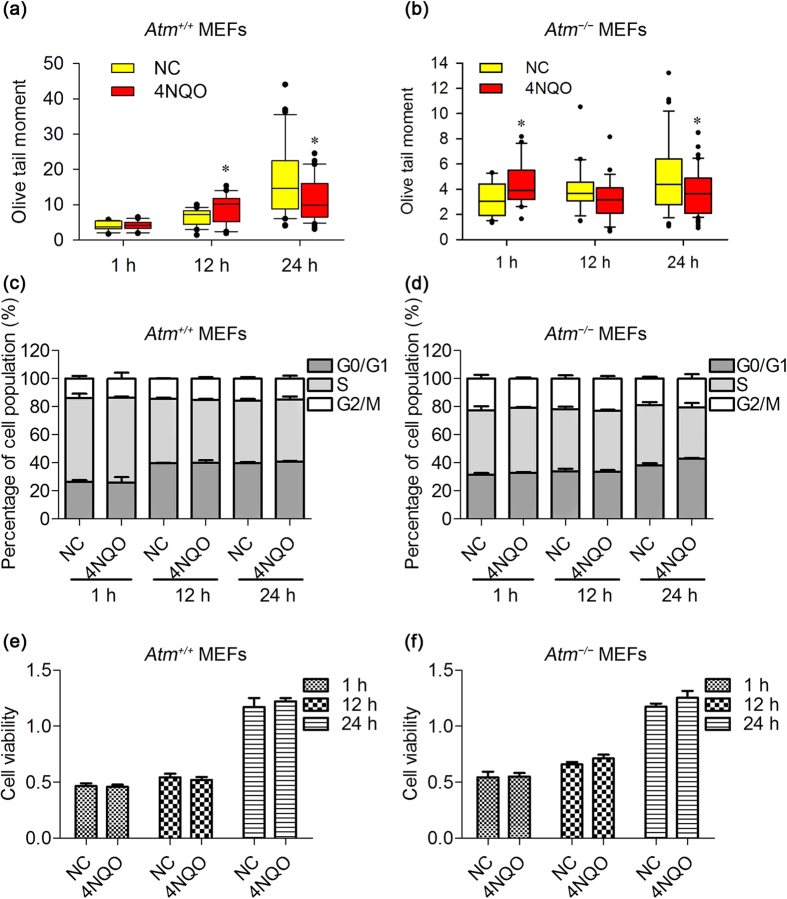
Effects of 0.005 μM 4-nitroquinoline 1-oxide (4NQO) on *Atm*^+/+^ and *Atm*^−/−^ MEFs. *Atm*^+/+^ and *Atm*^−/−^ MEFs were treated with 4NQO at 0.005 μM for 1, 12, and 24 h. (**a**,**b**) For the statistical analysis of DNA fragmentation (alkaline comet assay), values were quantified in 15 fields of view, with each field containing 10–20 cells. The experiments were repeated at least 3 times; (**c**,**d**) cell cycle progression, n = 3; and (**e**,**f**) cell viability, n = 3. The two-tailed paired Student’s *t*-test was used to determine the statistical significance of differences between the 4NQO treatment and negative control groups under the same experimental conditions. A probability level of *P* < 0.05 was considered statistically significant. *P < 0.05.

4NQO is a genotoxic agent that is often used as a positive control when detecting DNA damage. To explain the surprising phenomenon that we observed in this study, we gradually lowered the concentration of 4NQO and detected the same endpoints using the same methods. Interestingly, we obtained very similar results in both *Atm*^+/+^ and *Atm*^−/−^ MEFs when the concentration of 4NQO was sufficiently low (Fig. 7). Although 0.05 μM 4NQO produced first an increase and then a decrease in DNA damage, this concentration induced significant cell cycle progression arrest and cell viability inhibition (Supplementary Fig. S6). However, when the concentration was lowered to 0.005 μM, we observed not only the initial increase and then decrease in DNA damage but also no significant changes in either cell cycle progression or cell viability (Fig. 7). A similar effect was observed in low-dose H_2_O_2_ treatment (Supplementary Figs S8 and S9). In other words, even a well-recognised genotoxic substance could induce the same phenomenon that we revealed in this study when its concentration was sufficiently low.

## Discussion

To clarify whether RF-EMFs are carcinogenic to humans, it is necessary to determine whether they are genotoxic. As RF-EMFs are considered to be weak environmental factors that cannot break the chemical bonds in cells, and a large number of studies have provided inconsistent results regarding their genotoxicity in normal cells, it is reasonable to explore the impacts of RF-EMF exposure on genomic integrity in certain DNA-repair-deficient cell models. In this study, we adopted *Atm*^−/−^ MEFs as a model and found that 1,800 MHz RF-EMF exposure induced both SSBs and DSBs when the cells were exposed to it for 12 h. Consequently, the DNA damage repair mechanisms were activated, resulting in decreased DNA fragmentation levels, even lower than the background level in the cells. However, we only observed SSBs in *Atm*^+/+^ MEFs after 1 h of exposure, which might have been due to the presence of a normal DSB repair system; this type of damage could also be repaired. Our data suggest that 1) 1,800 MHz RF-EMF exposure can induce DNA damage after a short period of exposure, such as 1 h in *Atm*^+/+^ MEFs and 12 h in *Atm*^−/−^ MEFs; 2) the DNA damage effect depends on the cell type (in *Atm*^+/+^ MEFs, the main type of damage was SSBs, whereas in *Atm*^−/−^ MEFs, both SSBs and DSBs occurred); and 3) weak DNA damage can be repaired to a level even lower than in the control. The effect of an initial increase and then a decrease in DNA damage is very similar to the general hormetic dose response of a toxic substance.

Hormesis has traditionally been recognised as an unconventional dose-response relationship characterised by a biphasic response at low and high doses of a chemical, biological molecule, physical stressor, or other initiator of a response^19^. Hormesis usually presents as low-dose stimulation and high-dose inhibition^19^. Temporal features also play important roles because many studies show that the hormetic dose-response relationship can occur after an initial disruption in homeostasis^20^,^21^. Mechanistically, the disruption triggers an overcompensation response, which results in a hormetic effect at the late stage^20^. In our study, the initial increase in DNA damage would disrupt cellular homeostasis. To re-establish homeostasis, cells activate DNA repair mechanisms, and overcompensation stimulation could explain the subsequently observed level of DNA damage being lower than that in the control in the RF-EMF-exposed *Atm*^+/+^ and *Atm*^−/−^ MEFs. As this study is the first to report such a unique effect of RF-EMF, we would like to call it a hormesis-like effect.

To further support the idea that overcompensation is the basic mechanism of our observed hormesis-like effect and to reveal the kinetics of DNA damage and repair in the cells, we also exposed both MEF cell types to RF-EMF for only 1 h, then withdrew the exposure and continuously cultured the cells for another 23 or 35 h. DNA fragmentation in 1-h-exposed *Atm*^+/+^ MEFs was lower than the corresponding control after 23 h of normal culture but higher than after 35 h of normal culture (Supplementary Fig. S10). This observation is in line with our hypothesis, namely, the 1-h exposure induced DNA damage, thus disrupting cell homeostasis. The subsequently overcompensated DNA repair produced excessive DNA repair in the cells, resulting in subsequently lower-than-control DNA damage levels in the 23-h cells. Moreover, because cell homeostasis was already broken, culture stress caused more DNA damage in the 35-h cells. In *Atm*^−/−^ MEFs, although we did not detect significant DNA damage in RF-EMF-exposed cells, the 1-h RF-EMF treatment might still alter the kinetics of DNA damage induction and repair, presenting as a higher DNA fragmentation at 23 h and no significant difference at 35 h (Supplementary Fig. S10). It would certainly be interesting to further monitor the DNA damage and repair kinetics following 12-, 24-, and even 36-h exposure times by allowing the cells to repair in medium without RF-EMF exposure. Unfortunately, due to the limitations of cell culture, we could not perform this type of experiment. Based on our data, we suggest that the hormesis-like effect of RF-EMF depends on cell type and exposure duration.

To date, no reports of a hormetic effect have been published in the field of RF-EMF research. However, adaptive responses (ARs) have been documented in human blood lymphocytes, in tumour cells, and in animals after pre-exposure to low-intensity RF-EMF^22^,^23^. AR refers to a phenomenon in which pre-exposure to a low dose (adaptive dose) of a damaging agent induces significant resistance to subsequent high-dose (challenge dose) treatment^24^. The principal mechanism of AR is that a low-dose toxin or stressor activates or upregulates certain existing biological pathways, which in turn enhance the capacity of the organism to withstand more severe insults. Although the underlying mechanisms might differ between AR and hormesis, the dose ranges of the two effects are very similar, and some scientists have proposed that AR is a specific case of hormesis^25^. Regardless, the AR phenomenon and our findings suggest that there are new issues needing to be addressed in terms of the biological effects and health risk assessment of RF-EMFs, for which further studies are warranted.

Although AR is beneficial for biological systems exposed to RF-EMFs, the hormesis-like effect might have different consequences in these systems^21^. It is well accepted that a certain level of DNA damage is necessary for normal biological processes. For example, during DNA replication and transcription, DSBs generated by topoisomerases are essential for resolving topological problems^26^. In addition, during meiosis, the evolutionarily conserved enzyme Spo11 introduces numerous DSBs across the genome to achieve accurate meiotic chromosome segregation^27^. Therefore, an amount of DNA damage lower than the background level might not be advantageous. On the one hand, reduced DNA damage could protect the organism from potential harmful effects induced by an RF-EMF upon a short period of exposure, as we observed no influence on cell cycle progression or cell viability after a 36-h exposure. On the other hand, it is possible that a level of DNA damage lower than the background would impede normal cellular events if essential DSBs are not produced or are repaired too quickly due to the overcompensating DNA repair capacity, especially over the long term. It is worth pointing out that even well-known toxins, such as 4NQO and H_2_O_2_, could induce DNA damage hormesis without affecting cell behaviour.

RF-EMFs have been categorised as 2B human carcinogens by the IARC, but the debate on whether they are harmful is still ongoing. Vijayalaxmi and Prihoda analysed the INTERPHONE data and found decreased risks in 24.3% of studies and increased risks in only 0.7% of studies for meningioma, and 22.1% vs. 6.6% for glioma^28^. Furthermore, they proposed that the observed consistent and replicated pattern of reduced risk would be very unlikely to have occurred just by chance and that RF-EMF-induced AR might account for this phenomenon^28^. However, their conclusion could not explain the increased risks of both tumours. To fully understand the consequences of AR or a hormesis-like effect induced by RF-EMFs, we need to explore the long-term effects of RF-EMF exposure in cells, animals, and human populations and to elucidate their underlying mechanisms.

In conclusion, we report a hormesis-like effect of 1,800 MHz RF-EMF exposure on DNA damage in *Atm*^+/+^ and *Atm*^−/−^ MEFs. Although reduced DNA damage might help to transiently maintain genomic integrity in response to RF-EMF exposure, the long-term biological consequences and health impacts need to be addressed.

## Methods

### Cell lines and cultures

Spontaneously immortalised *Atm*^+/+^ and *Atm*^−/−^ MEFs were obtained from Chen D. J. (Lawrence Berkeley National Laboratory, Berkeley, California, USA)^29^. The cells were cultured in Alpha Minimum Essential Medium (HyClone; Thermo Scientific, Shanghai, China) supplemented with 10% (v/v) heat-inactivated foetal bovine serum (HyClone) at 37 °C in a humidified atmosphere containing 5% CO_2_.

### Exposure system

The RF-EMF exposure system was built and provided by the Foundation for Information Technologies in Society (IT’IS Foundation, Zurich, Switzerland), as described previously^7^. Briefly, the system is based on a dual-resonant R18-waveguide setup and holds six 35-mm Petri dishes positioned in the H-field maximum of the standing wave. The waveguides can be switched on for RF-EMF exposure or off for sham exposure. The RF-EMF signal mimics the basic pulse structure of the Global System for Mobile Communication signal at a frequency of 1,800 MHz. Both of these waveguides are placed inside a CO_2_ incubator (Hera Cell 240; Thermo Scientific, Waltham, MA, USA). The SAR value and temperature in the waveguide chambers are monitored using a computer.

### Cell exposure protocol

The *Atm*^+/+^ and *Atm*^−/−^ MEFs were sub-cultured by seeding 1 × 10^5^ cells in each 35-mm Petri dish (Nunc; Thermo Scientific, Shanghai, China). Twelve hours after seeding, the cultures were intermittently (5 min on/10 min off) exposed or sham exposed to a 1,800 MHz RF-EMF at an average SAR of 4.0 W/kg for 1, 12, 24, or 36 h. The intermittent exposure protocol mimics the daily life exposure scenario of mobile phones.

For the γH2AX focus formation assay, the cells were seeded in 35-mm Petri dishes, each containing a glass coverslip (Thermo Fisher Scientific, Shanghai, China) on the bottom.

During exposure, the temperature difference between the RF-EMF- and sham-exposed chambers did not exceed 0.1 °C.

### Immunofluorescence staining of γH2AX

Immunofluorescent detection of γH2AX foci was performed as previously described^7^. Immediately after exposure, the cells on the coverslips were fixed in a 4% paraformaldehyde solution for 15 min at 4 °C and were then permeabilised in 0.5% Triton X-100 at 4 °C for 15 min. Non-specific binding was blocked with blocking serum (Zhongshan Golden Bridge Biotechnology Co., Ltd., Beijing, China) for 2 h, and incubation with a primary anti-γH2AX antibody (diluted 1:1,000; Upstate, Millipore, Temecula, CA) for 2 h was followed by incubation with a tetramethyl rhodamine isothiocyanate-conjugated goat anti-mouse secondary antibody (diluted 1:300; Zhongshan Goldenbridge Biotechnology Co., Ltd.) for 1 h at room temperature. Thereafter, the samples were stained with 1.0 μg/mL 4′,6-diamidino-2-phenylindole (DAPI; Sigma-Aldrich, St. Louis, MO, USA) for 15 min. Each step was followed by 3 washes for 5 min each in phosphate-buffered saline. Finally, the coverslip was removed from the Petri dish and mounted onto a glass slide for visualisation.

The γH2AX foci in the nuclei were observed using an Olympus AX70 fluorescence microscope (Olympus, Tokyo, Japan) with a 60× oil immersion objective. Approximately 100 cells from 4–6 fields of view of each slice, three slices per experiment, were randomly photographed and analysed. The average number of γH2AX foci per cell was used as an index to evaluate γH2AX focus formation. All of the experiments were repeated at least 3 times.

### Alkaline and neutral comet assays

Cellular DNA fragments were detected by single-cell gel electrophoresis, i.e., the comet assay, as described in detail elsewhere^7^. Briefly, immediately after exposure, the cells were harvested, mixed with 0.65% low-melting agarose and added onto slides pre-coated with 0.65% normal-melting agarose. The cells were then denatured with ice-cold lysis buffer [2.5 M NaCl, 1% sodium N-lauroyl sarcosinate, 0.1 M Na_2_-ethylenediaminetetraacetic acid (EDTA), 10 mM Tris-HCl, pH 10.0] containing 1% Triton X-100 for 1 h. Then, for the alkaline comet assay, cells were treated with 0.5 mg/mL DNase-free proteinase K (Amresco, OH, USA) in lysis buffer without Triton X-100 for 2 h at 37 °C; for the neutral comet assay, cells were treated with 10 μg/mL ribonuclease A (RNase A; Thermo Scientific, Shanghai, China) after lysis and then with 0.5 mg/mL DNase-free proteinase K in lysis buffer without Triton X-100 for 2 h at 37 °C. After the DNA had unwound in the electrophoresis solution (alkaline: 0.3 M NaOH, 0.1% 8-hydroxyquinoline, 2% dimethylsulfoxide, 10 mM tetrasodium EDTA, pH 13; neutral: 100 mM Tris, 300 mM sodium acetate, and acetic acid, pH 9.0) for 20 min, the slices were electrophoresed at a constant current of 300 mA (20 V, 0.4 V/m) for 20 min. At the end of the electrophoresis, the slices were neutralised in Tris buffer (0.4 M, pH 7.5) for 5 min and stained with Gel-Red (Biotium, Hayward, CA, USA). The DNA “comets” were visualised with a 10× lens fitted onto a fluorescence microscope (Nikon, Tokyo, Japan). Approximately 100 comets were randomly photographed from 6–8 fields of view of each slice, three slices per experiment, and the DNA damage parameters were analysed using CASP 1.2.2 software (Krzysztof Konca, Wroclaw, Poland). The Olive tail moment and percentage of tail DNA for each comet were calculated. All of the experiments were repeated at least 3 times.

### Western blot analysis

Western blotting was performed as previously described^30^, with minor modifications. Cells were lysed with radioimmunoprecipitation assay (RIPA) lysis buffer consisting of 150 mM NaCl, 10 mM Tris, pH 7.5, 1% NP40, 1% deoxycholate, 0.1% sodium dodecyl sulfate (SDS), and 1× protease inhibitor cocktail (Roche Diagnostics, Indianapolis, IN, USA). Cell lysates were separated by electrophoresis on 10% SDS-polyacrylamide gels, transferred to nitrocellulose membranes (Whatman, Freiburg, Germany), blocked with 3% bovine serum albumin in Tris-buffered saline and Tween 20, and blotted with the primary antibody followed by the IRDye 800CW-labelled goat anti-rabbit secondary antibody (Li-COR Biosciences, Lincoln, NE, USA). The blot was visualised using an Odyssey Infrared Imaging System (Li-COR) and was then quantified using densitometry. The primary rabbit anti-XRCC1 (ab1947, 1:2,000) and anti-phospho-XRCC1 (ab195205, 1:2,000) antibodies were from Abcam (Shanghai, China); the mouse anti-β-actin antibody (sc47778, 1:2,000) was from Santa Cruz Biotechnology (Santa Cruz, CA, USA).

### Cell cycle analysis

After exposure, the cells were harvested, resuspended in pre-cooled 70% ethanol, and stored at −20 °C overnight. The cells were stained with 50 μg/mL propidium iodide (Sigma-Aldrich) diluted in phosphate-buffered saline containing 50 μg/mL RNase A at room temperature for 30 min. Then, the cell cycle distribution was analysed by flow cytometry (FC500MCL; Beckman Coulter, CA, USA), in which 1 × 10^4^ events per sample were acquired, and the proportions of cells in the G_0_/G_1_, S, and G_2_/M phases were determined using Wincycle32 software (Beckman Coulter).

### Cell viability analysis

For RF-EMF exposure, 1 × 10^5^ cells per dish were seeded in 35-mm Petri dishes 12 h before exposure. After exposure, the cells were detached using 200 μL of 0.25% trypsin-EDTA and were resuspended in 500 μL of culture medium. Then, 2 × 10^4^ cells per well were re-seeded into a 96-well plate. After 6 h of culture, cell viability was determined using a Cell Counting Kit-8 (CCK-8; Dojindo Molecular Technologies, Inc., Kumamoto, Japan).

For 4NQO treatment, 1 × 10^4^ cells per well were seeded directly into a 96-well plate. After 12 h of incubation, the cells were treated with different concentrations of 4NQO for 1, 12, and 24 h.

For cell viability measurements, 10 μL of CCK-8 reagent was added into each well of a 96-well plate with cultured cells, and the plate was incubated for an additional 2 h at 37 °C. The optical density value of each well was measured using a microplate reader (Varioskan Flash; Thermo Scientific, Waltham, MA, USA) with a test wavelength of 450 nm.

## Additional Information

**How to cite this article**: Sun, C. *et al*. Mobile phone signal exposure triggers a hormesis-like effect in *Atm*^+/+^ and *Atm*^−/−^ mouse embryonic fibroblasts. *Sci. Rep.* **6**, 37423; doi: 10.1038/srep37423 (2016).

**Publisher's note**: Springer Nature remains neutral with regard to jurisdictional claims in published maps and institutional affiliations.

## Supplementary Material

Supplementary Material

Supplementary Tables
