# Supplementary material for: Mobile phone signal exposure triggers a hormesis-like effect in Atm+/+ and Atm−/− mouse embryonic fibroblasts
Source: Sci Rep. 2016 Nov 18;6:37423. doi: 10.1038/srep37423 (PMC5114646; doi:10.1038/srep37423)
Supplement: Supplementary Material [file srep37423-s1.pdf]

Mobile phone signal exposure triggers a hormesis-like effect in *Atm*$^{+/+}$ and *Atm*$^{-/-}$ mouse embryonic fibroblasts

Chuan Sun[1], Xiaoxia Wei[1], Yue Fei[1], Liling Su[1], Xinyuan Zhao[1], Guangdi Chen[1,3,*] and Zhengping Xu[1,2,3,*]

[1] Bioelectromagnetics Laboratory, Zhejiang University School of Medicine, Hangzhou 310058, China

[2] Collaborative Innovation Center for Diagnosis and Treatment of Infectious Diseases, Zhejiang University, Hangzhou 310003, China

[3] Institute of Environmental Health, Zhejiang University School of Public Health, Hangzhou 310058, China

*Correspondence to Dr. Zhengping Xu or Dr. Guangdi Chen, 866 Yuhangtang Road, Bioelectromagnetics Laboratory, Zhejiang University School of Medicine, Hangzhou 310058, China; Tel: +86-571-88208008; Fax: +86-571-88208163; Email: zpxu@zju.edu.cn or chenguangdi@zju.edu.cn.

Materials and methods

Cell culture

For hydrogen peroxide (H_2O_2) treatment, 5×10^4 cells per well were seeded in 12-well plates 12 h before treatment.

Cell counting analysis

After treatment, cells were visualized with a 20 × lens fitted onto a microscope (Nikon, Tokyo, Japan). For each sample, the number of cells was calculated by counting cells in 5 randomly captured images.

Cell apoptosis analysis

Annexin V/ propidium iodide (PI) apoptosis kit (MultiSciences Biotech, Hangzhou, China) was applied to determine the cell apoptosis rate. After the radiofrequency electromagnetic fields (RF-EMF) exposure or H_2O_2 treatment, both floating and attached cells were harvested. Five μL annexin V-FITC and 10 μL of PI from the kit were used to stain the apoptotic cells for 5 min in the dark, and then the cells were analyzed by flow cytometry (FC500MCL; Beckman Coulter, CA, USA).

5-ethynyl-2'-deoxyuridine staining analysis

Click-iT® Plus EdU Alexa Fluor® 488 Flow Cytometry Assay Kit (Invitrogen, Carlsbad, CA) was applied to detect EdU incorporation. After RF-EMF exposure, cells in dishes were incubated with 50 μM 5-ethynyl-2'-deoxyuridine (EdU) for 30 min. Then, cells were harvested and fixed for EdU staining following the manufacturer’s instructions. The results were analysed by flow cytometry (FC500MCL; Beckman Coulter).

Figure legends

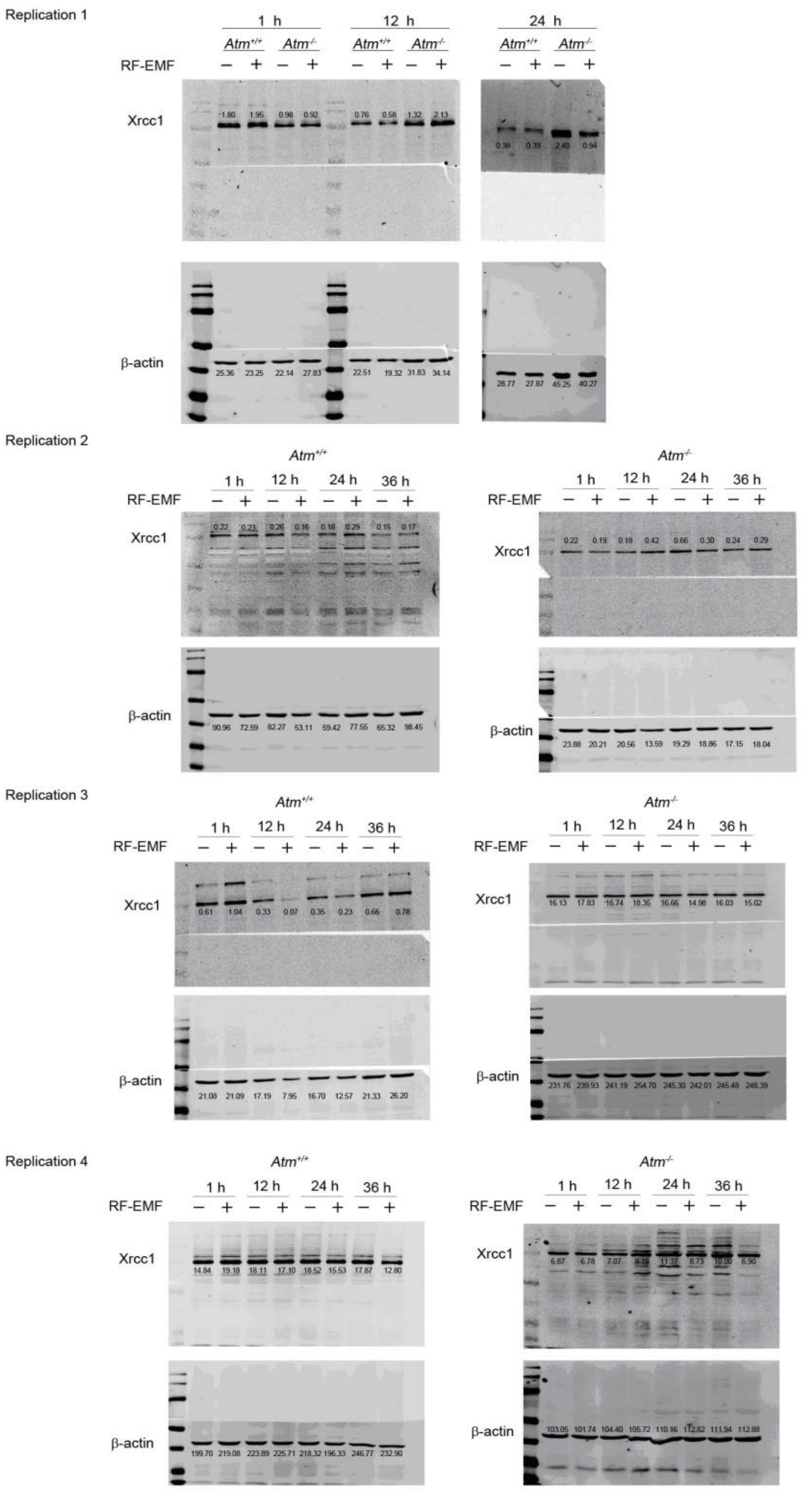

Figure S1. The blots used for quantifying Xrcc1 level in *Atm*$^{+/+}$ and *Atm*$^{-/-}$ MEFs. Images of Western blotting show the levels of Xrcc1 and β-actin in *Atm*$^{+/+}$ and *Atm*$^{-/-}$ MEFs after sham exposure or exposure to 1,800 MHz RF-EMF at 4.0 W/kg for up to 36 h.

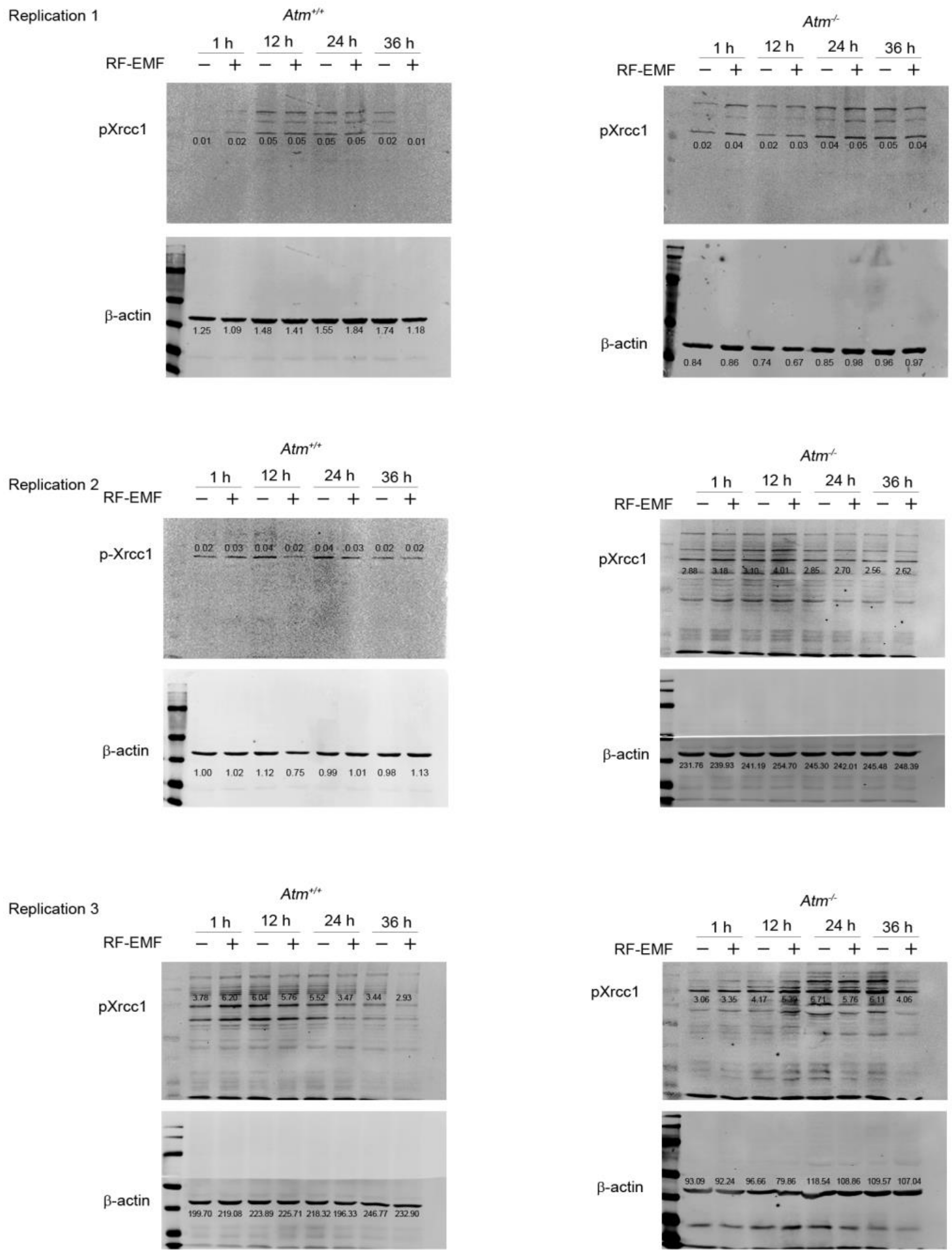

Figure S2. The blots used for quantifying p-Xrcc1 level in *Atm*$^{+/+}$ and *Atm*$^{-/-}$ MEFs. Images of Western blotting show the levels of phospho-Xrcc1 (p-Xrcc1) and β-actin in *Atm*$^{+/+}$ and *Atm*$^{-/-}$ MEFs after sham exposure or exposure to 1,800 MHz RF-EMF at 4.0 W/kg for up to 36 h.

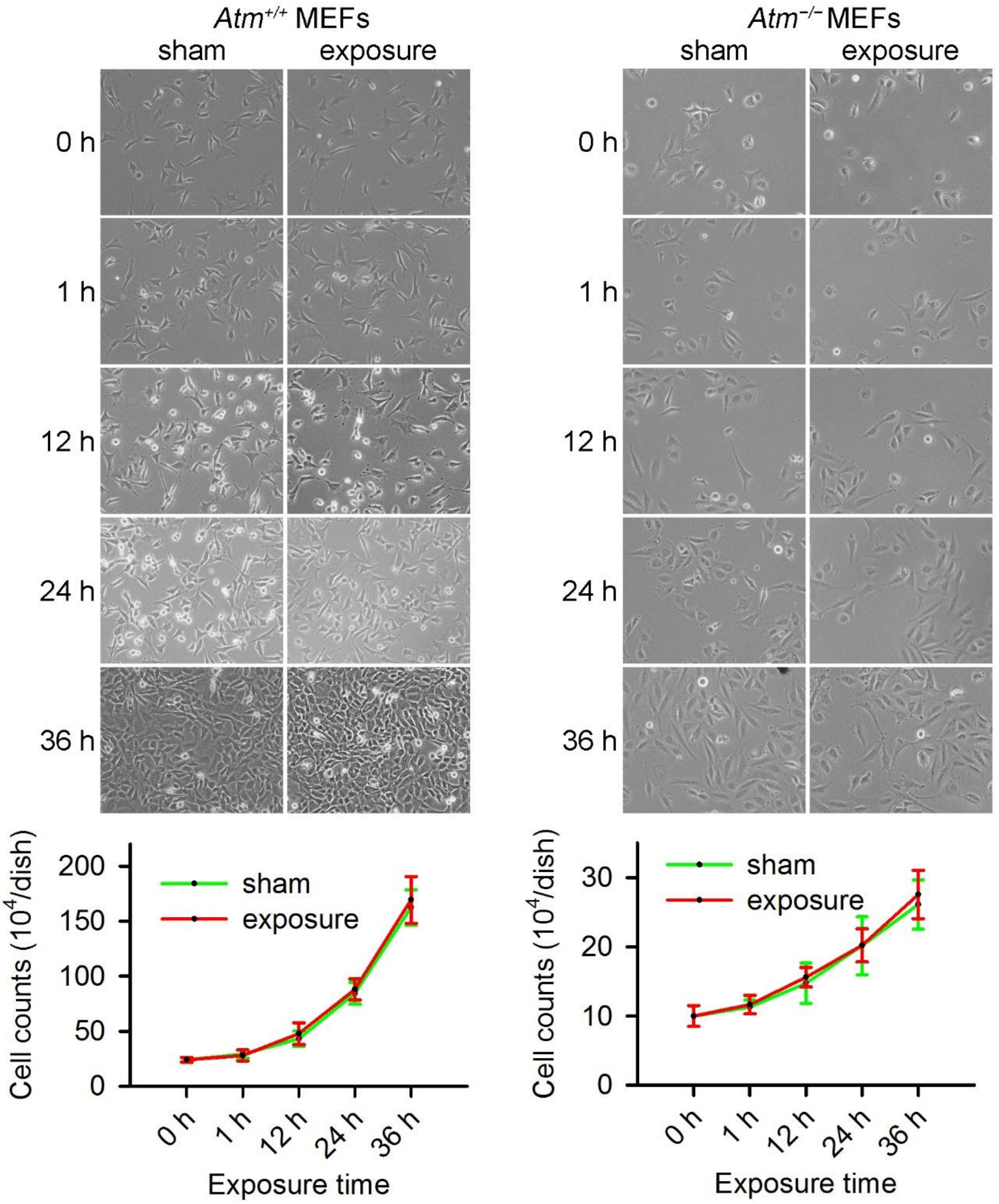

Figure S3. The effect of 1,800 MHz RF-EMF exposure on cell proliferation in *Atm*$^{+/+}$ and *Atm*$^{-/-}$ MEFs. Cell densities were measured in *Atm*$^{+/+}$ and *Atm*$^{-/-}$ MEFs after exposure to 4.0 W/kg 1,800 MHz RF-EMF for up to 36 h. Graphs show the cell counts of each group. Values shown are means ± SEM; n = 5. Two-tailed paired Student's *t*-test was used to determine the statistical significance of differences between the RF-EMF exposure and sham exposure groups. A probability level of $P < 0.05$ was considered statistically significant.

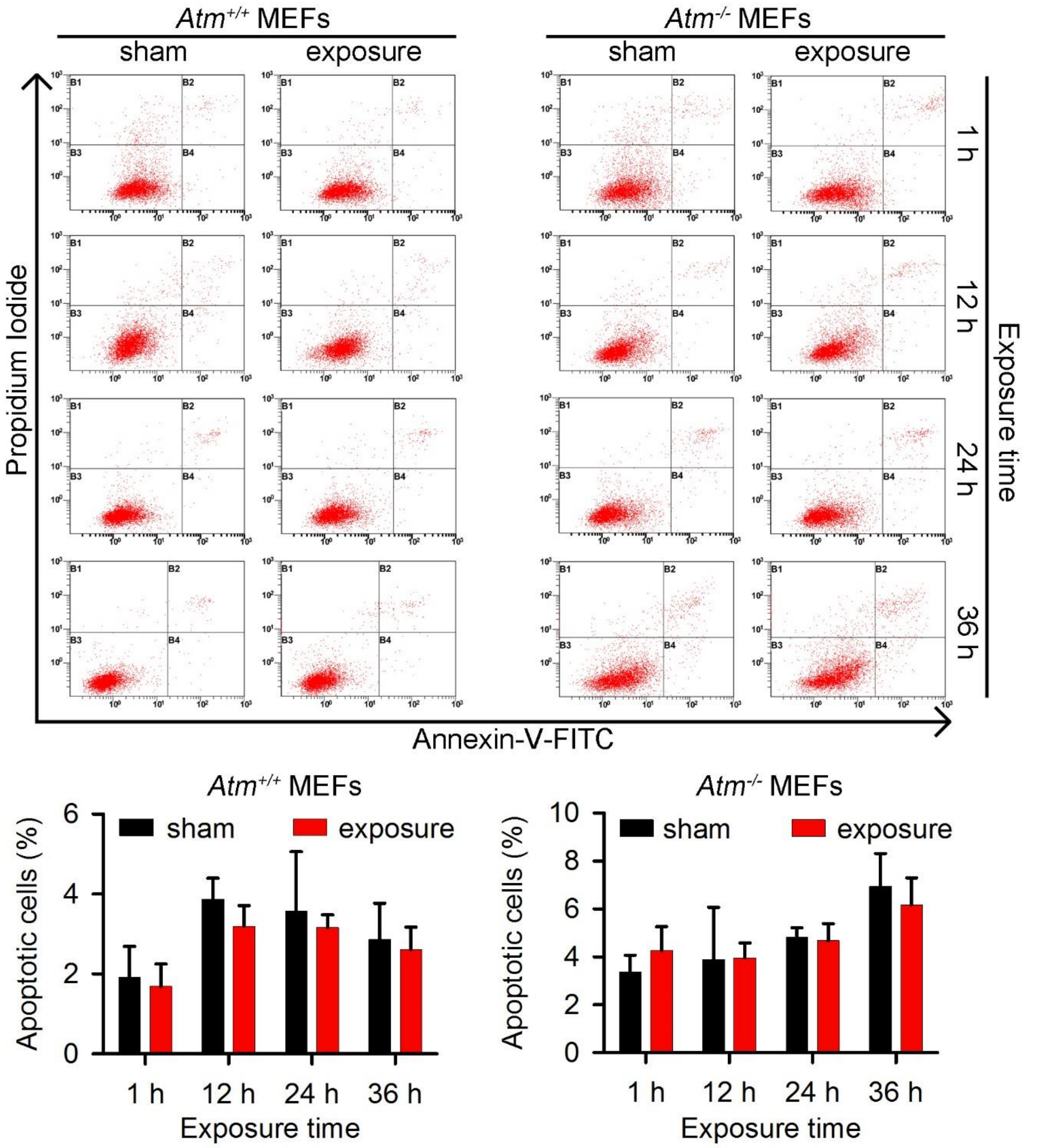

Figure S4. The effect of 1,800 MHz RF-EMF exposure on apoptosis in *Atm*$^{+/+}$ and *Atm*$^{-/-}$ MEFs. Apoptosis was analysed in *Atm*$^{+/+}$ and *Atm*$^{-/-}$ MEFs after exposure to 4.0 W/kg of 1,800 MHz for up to 36 h. Histograms show the percentage of apoptotic cells in each group. Values shown are means ± SEM; n = 3. Two-tailed paired Student's *t*-test was used to determine the statistical significance of differences between the RF-EMF exposure and sham exposure groups. A probability level of $P < 0.05$ was considered statistically significant.

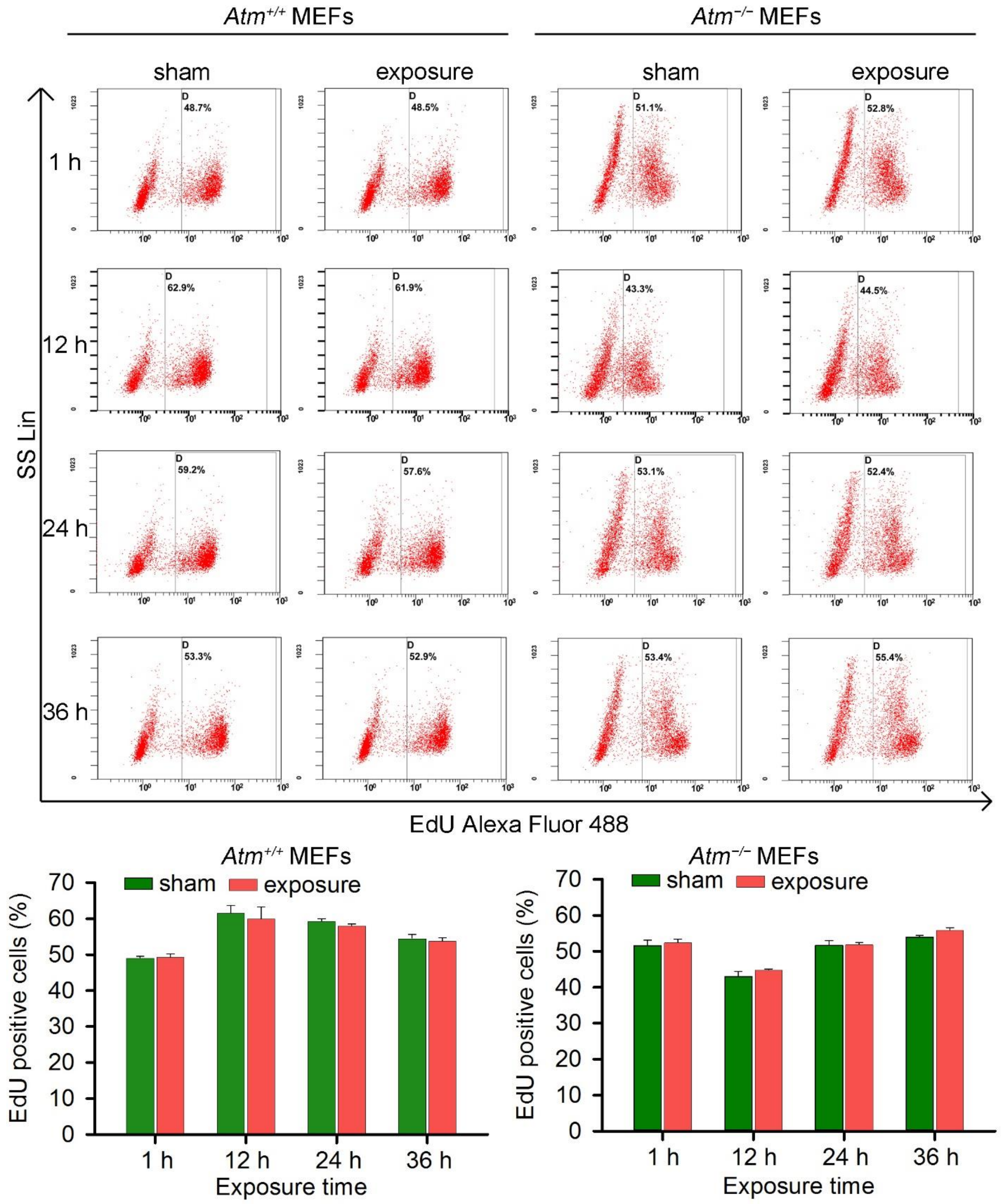

Figure S5. The effect of 1,800 MHz RF-EMF exposure on DNA synthesis in *Atm*$^{+/+}$ and *Atm*$^{-/-}$ MEFs. EdU staining was conducted in *Atm*$^{+/+}$ and *Atm*$^{-/-}$ MEFs after exposure to 4.0 W/kg of 1,800 MHz RF-EMF for up to 36 h. Histograms show the percentage of EdU positive cells in each group. Values shown are means ± (standard error mean) SEM; n =3. Two-tailed paired Student's *t*-test was used to determine the statistical significance of differences between the RF-EMF exposure and sham exposure groups. A probability level of $P < 0.05$ was considered statistically significant.

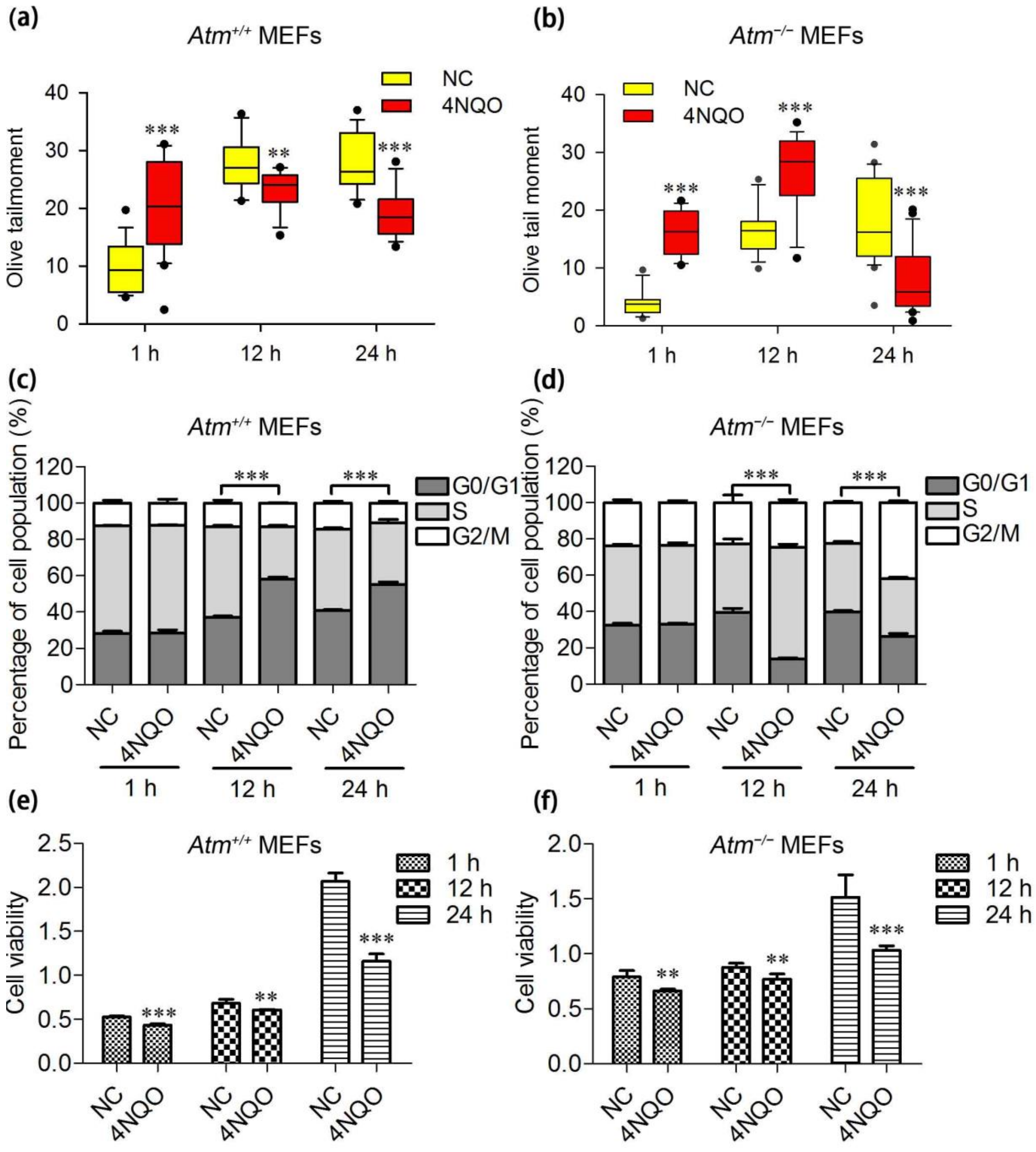

Figure S6. Effect of 0. 05 µM 4-nitroquinoline 1-oxide (4NQO) on *Atm*$^{+/+}$ and *Atm*$^{-/-}$ MEFs. *Atm*$^{+/+}$ and *Atm*$^{-/-}$ MEFs were treated with 4NQO at 0.05 µM for 1, 12, and 24 h. (**a**, **b**) Statistical analyses of the DNA fragmentation (alkaline comet assay), values were quantified in 15 view fields, each field contained 10-20 cells, and the experiments were repeated at least 3 times; (**c**, **d**) cell cycle progression, n = 3; and (**e**, **f**) cell viability, n = 3. Two-tailed paired Student's *t*-test was used to determine the statistical significance of differences between 4NQO treatment and negative control group under same experimental conditions. A probability level of $P < 0.05$ was considered statistically significant. $*P < 0.05$, $**P < 0.01$, $***P < 0.001$.

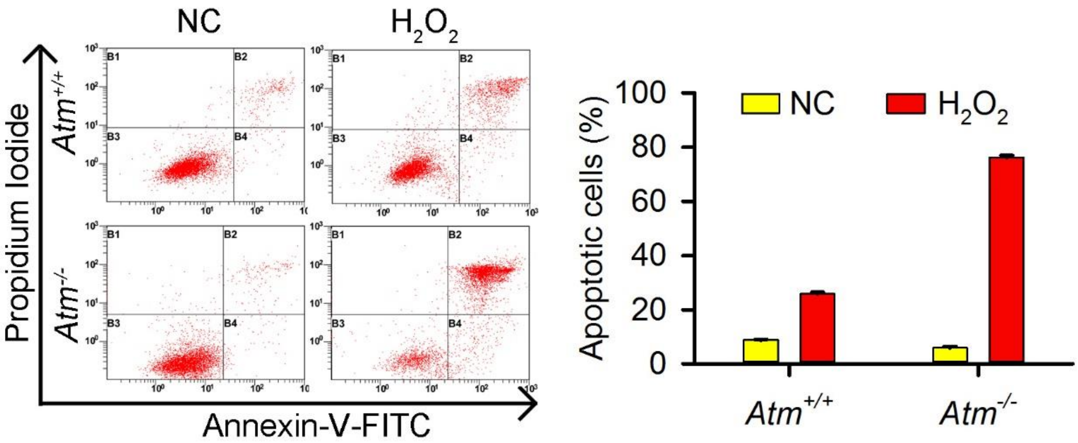

Figure S7. The effect of H_2O_2 on apoptosis in *Atm*$^{+/+}$ and *Atm*$^{-/-}$ MEFs. Apoptosis was analysed in *Atm*$^{+/+}$ and *Atm*$^{-/-}$ MEFs after treated with or without 200 μM H_2O_2 for 2 h. Histogram shows the percentage of apoptotic cells in each group. Values shown are means ± standard error mean (SEM); n = 3.

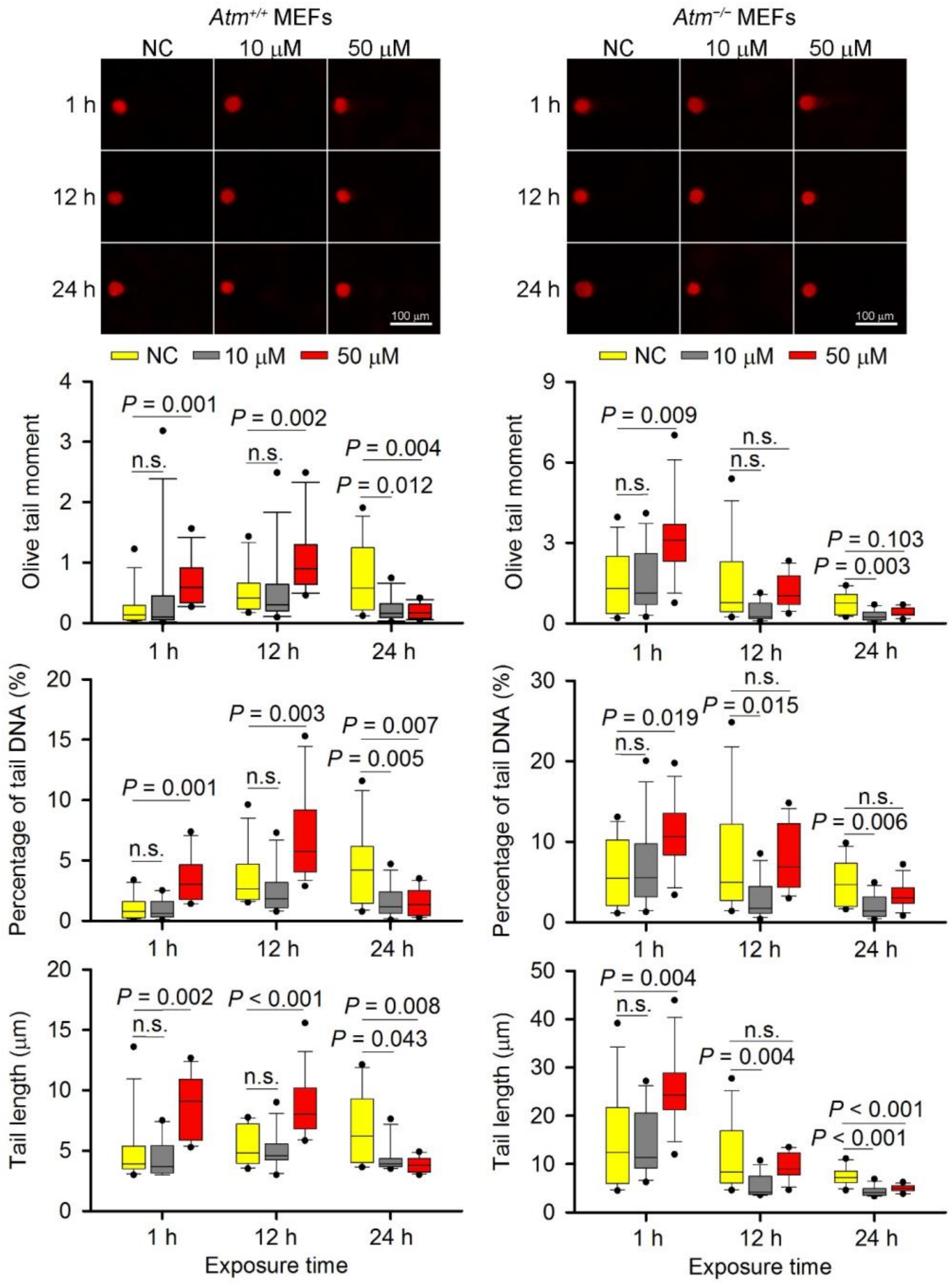

Figure S8. The effect of H_2O_2 on DNA fragmentation in *Atm*$^{+/+}$ and *Atm*$^{-/-}$ MEFs. *Atm*$^{+/+}$ and *Atm*$^{-/-}$ MEFs were treated with 0 (NC), 10, or 50 μM H_2O_2 for up to 24 h, and then DNA fragmentation was analyzed by alkaline comet assay. Boxplots show the quantified data of DNA fragmentation in each group; values were quantified in 15 view fields, each field contained 10–20 cells, and the experiments were repeated at least 3 times. Mann-Whitney Rank Sum Test was applied to determine the statistical significance of differences between H_2O_2 treatment and negative control group under same experimental conditions. A probability level of $P < 0.05$ was considered statistically significant. Abbreviation: n.s., no significance.

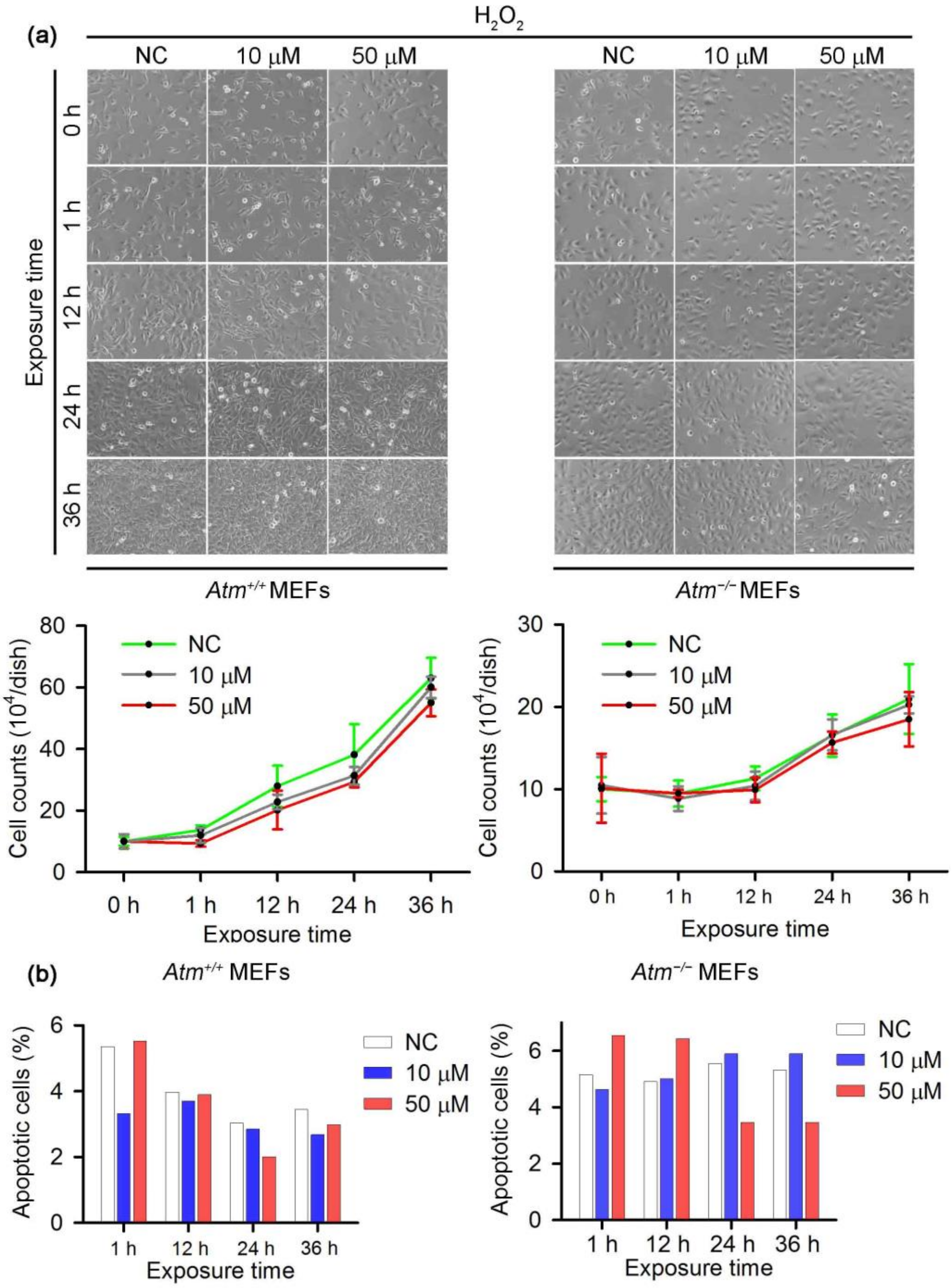

Figure S9. The effect of H_2O_2 on cell proliferation and apoptosis in *Atm*$^{+/+}$ and *Atm*$^{-/-}$ MEFs. *Atm*$^{+/+}$ and *Atm*$^{-/-}$ MEFs were treated with 0 (NC), 10, or 50 μM H_2O_2 for up to 36 h. (**a**) Representative images and graphs show the cell densities of each group. Values shown are means ± SEM; n = 4. (**b**) Histograms show the percentage of apoptotic cells in each group. Two-tailed paired Student's *t*-test was used to determine the statistical significance of differences between H_2O_2 treatment and negative control group under same experimental conditions. A probability level of $P < 0.05$ was considered statistically significant.

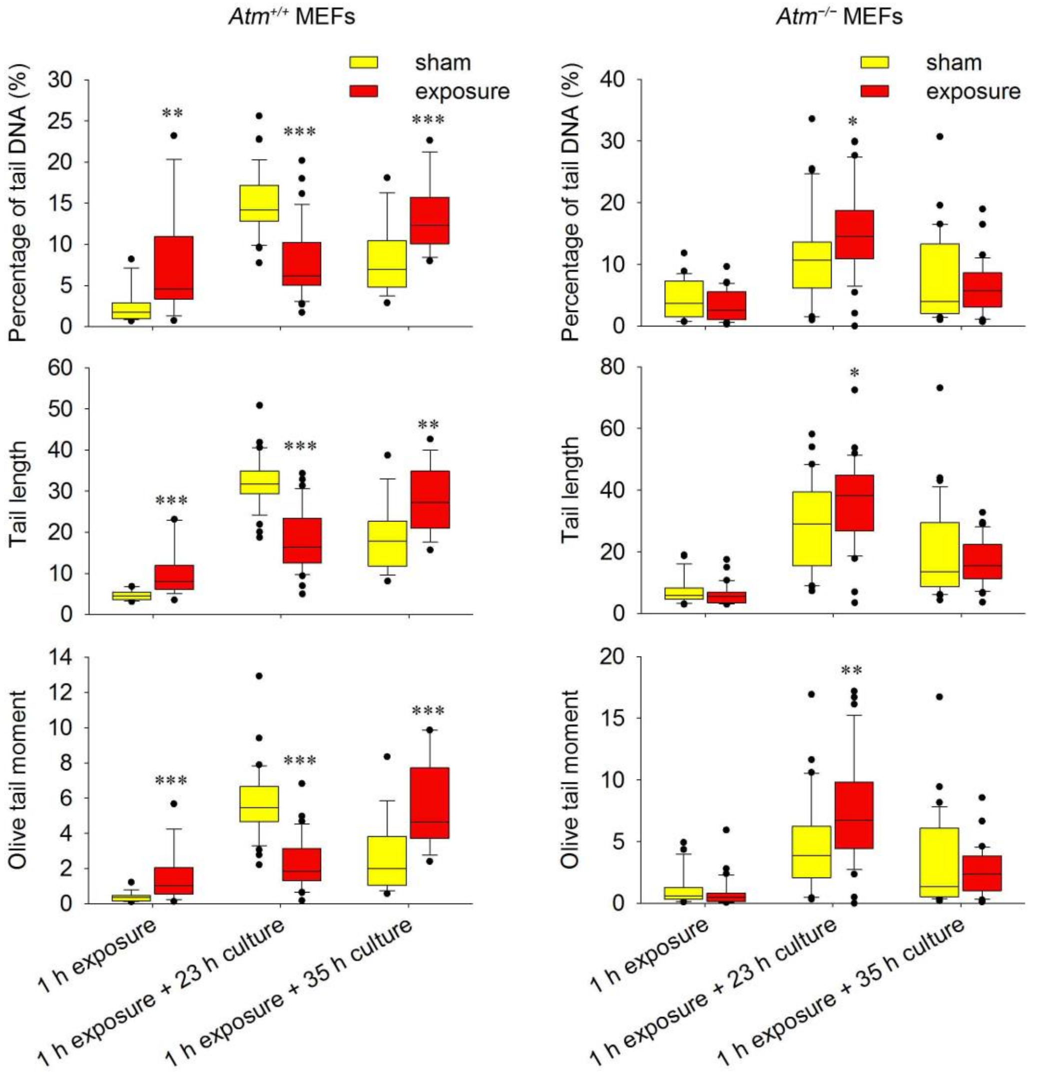

Figure S10. The dynamic changes of DNA fragmentation in ***Atm***$^{+/+}$ **and** ***Atm***$^{-/-}$ **MEFs at indicated time points following 1-hour-exposure to 1800 MHz RF-EMF.** *Atm*$^{+/+}$ and *Atm*$^{-/-}$ MEFs were exposed or sham-exposed to 1800 MHz RF-EMF at 4.0 W/kg for 1 hour and then normally cultured for another 23 or 35 hours. DNA fragmentation was detected by alkaline comet assay, and the percentage of tail DNA (%), tail length and olive tail moment were used to evaluate the level of DNA fragmentation in cells. Boxplots show the quantified data of DNA fragmentation in each group; values were quantified in 18 view fields, and each field contained 10-20 cells. The experiments were repeated 2 times for 1-hour exposure plus 23-hour normal culture and 3 times for 1-hour exposure plus 36-hour normal culture, and triplicate cultures were used for each experiment. Mann-Whitney Rank Sum Test was used to determine the statistical significance of differences between exposure and sham-exposure groups. A probability level of $P < 0.05$ was considered statistically significant. $*P < 0.05$, $**P < 0.01$, $***P < 0.001$.
